# Complex Spike Wars: a New Hope

**DOI:** 10.1007/s12311-018-0960-3

**Published:** 2018-07-07

**Authors:** Martha L. Streng, Laurentiu S. Popa, Timothy J. Ebner

**Affiliations:** Department of Neuroscience, University of Minnesota, Lions Research Building, Room 421, 2001 Sixth Street S.E, Minneapolis, MN 55455 USA

**Keywords:** Purkinje cell, Complex spike, Climbing fibers, Simple spike, Motor error, Cerebellar cortex

## Abstract

The climbing fiber–Purkinje cell circuit is one of the most powerful and highly conserved in the central nervous system. Climbing fibers exert a powerful excitatory action that results in a complex spike in Purkinje cells and normal functioning of the cerebellum depends on the integrity of climbing fiber–Purkinje cell synapse. Over the last 50 years, multiple hypotheses have been put forward on the role of the climbing fibers and complex spikes in cerebellar information processing and motor control. Central to these theories is the nature of the interaction between the low-frequency complex spike discharge and the high-frequency simple spike firing of Purkinje cells. This review examines the major hypotheses surrounding the action of the climbing fiber–Purkinje cell projection, discussing both supporting and conflicting findings. The review describes newer findings establishing that climbing fibers and complex spikes provide predictive signals about movement parameters and that climbing fiber input controls the encoding of behavioral information in the simple spike firing of Purkinje cells. Finally, we propose the dynamic encoding hypothesis for complex spike function that strives to integrate established and newer findings.

## Introduction

The distinctive morphology, cellular actions, and physiological properties of the climbing fiber–Purkinje cell synapse suggest a unique role in the cerebellum and behavior [1–3]. As one of the strongest synapses in the mammalian central nervous system, the action of climbing fibers on Purkinje cells sparked great interest among neuroscientists and continues to be at the center of efforts to understand cerebellar function. Climbing fiber input is essential to the cerebellum’s role in controlling movements, and malfunction of the climbing fiber–Purkinje cell synapse is a central component in the movement disorder characterizing several of the spinocerebellar ataxias [4]. Several major hypotheses have been proposed on the function of the climbing fiber–Purkinje cell synapse in both cerebellar information processing and behavior. This review examines each of these established hypotheses. The review also describes recent results that offer new insights into the action of the climbing fibers in the cerebellum.

## Properties of Climbing Fibers and Synaptic Action on Purkinje Cells

**Fig. 1:**
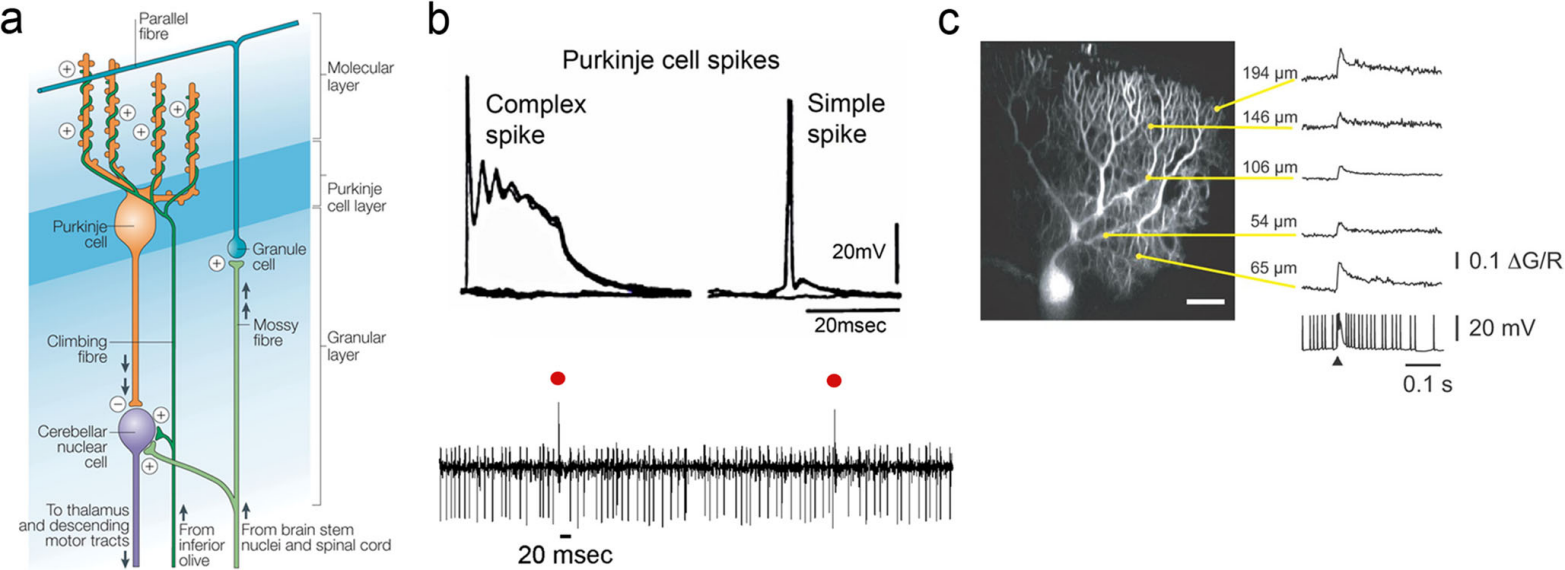
Circuitry of the cerebellum and synaptic action of the climbing fibers**. a** Canonical circuitry of the cerebellum including climbing fiber–Purkinje cell circuit, mossy fiber-granule cell-parallel fiber–Purkinje cell circuit and Purkinje cell projection to the cerebellar nuclei. “+” and “-” denote excitatory and inhibitory synapses, respectively (modified with permission from [31]). **b** Top panel: intracellular recording from a Purkinje cell of both a complex spike (CS) with its initial Na^+^ spike and prolonged depolarization and a spontaneous simple spike SS (modified with permission from [12]). Bottom panel: extracellular recording from a Purkinje cell shows the high-frequency SS firing and low-frequency CS discharge (red dots) (modified with permission from [61]). **c** Spatial profile of Ca^2+^ influx evoked by climbing fiber input displaying Ca^2+^ transients at multiple locations along the dendritic tree (modified with permission from [61])

Climbing fiber afferents originate solely from the inferior olive, a group of nuclei in the lower medulla, and form the olivocerebellar projection. As one of the most conserved pathways in the vertebrate nervous system, this highlights the importance of the olivocerebellar projection [5]. The axons from inferior olivary neurons cross in the midline and travel through the inferior cerebellar peduncle to enter the cerebellum. Climbing fibers provide one of the two main inputs to the cerebellar cortex, the other source being mossy fibers (Fig. 1a). Unique to the climbing fibers is that they monosynaptically innervate Purkinje cells, the sole output neuron of the cerebellar cortex. Therefore, climbing fibers have direct control over the output of the cerebellar cortex.

Through hundreds of glutamatergic synapses along two thirds of the proximal dendritic tree, a Purkinje cell receives input from a single climbing fiber in the adult animal (Fig. 1a). Each climbing fiber synapses on 5–10 Purkinje cells that tend to be located in a parasagittal plane, a prominent feature of the olivocerebellar projection. This parasagittal architecture matches the overall longitudinal zonation of the cerebellum. In this organization, a parasagittal zone or strip of Purkinje cells, as defined by the presence of zebrin II ± bands [6], receives climbing fiber input from a circumscribed region of the inferior olive, and the same zone of Purkinje cells project to a specific region of the cerebellar nuclei (for review, see [7–9]). The differential expression of numerous molecules on Purkinje cells within these zonal compartments complements the parasagittal anatomy (for review, see [6]).

The climbing fiber–Purkinje cell synapse is one of the most powerful in the CNS and its distinctive properties have stimulated intense interest [10–12]. Firing at low rates (~ 0.5–2.0/s), a climbing fiber produces a massive depolarization of the entire Purkinje cell resulting in a complex spike (CS) (Fig. 1b). A CS consists of a large Na^+^ somatic spike and burst of smaller spikelets generated in the initial axon segment. The strong depolarization also opens voltage-gated Ca^2+^ channels that result in Ca^2+^ spikes throughout the entire dendritic tree (Fig. 1c) [13, 14]. Therefore, while separate mechanisms underlie the CS and dendritic Ca^2+^ spikes, both have important roles in understanding climbing fiber action on Purkinje cells. Although traditionally considered an all-or-none response [1], recent work demonstrates that both CSs and dendritic Ca^2+^ responses can be graded via pre- and post-synaptic modulation (for review, see [15]).

In sharp contrast to the monosynaptic climbing fiber–Purkinje cell circuit, mossy fibers act through the highly divergent granule cell-parallel fiber network (Fig. 1a). Mossy fibers originate from a large number of sites including the spinal cord and brainstem nuclei, with a large projection from the cerebral cortex via the pons [1, 2]. A Purkinje cell receives input from over 100,000 parallel fibers that modulate the intrinsically driven high-frequency simple spike (SS) discharge [1, 16]. Running transversely along a folium, an individual parallel fiber synapses on several hundred Purkinje neurons but makes only a few *en-passant* synapses on an individual Purkinje cell [1, 2]. In comparison with climbing fiber input, a parallel fiber produces only a small excitatory response in a Purkinje cell [17].

To complete the circuitry, Purkinje cells project to and inhibit the cerebellar and vestibular nuclei. In turn, a population of excitatory neurons in the cerebellar and vestibular nuclei project to the spinal cord, brainstem, and thalamic nuclei, modulating downstream structures including the cerebral cortex via the cerebello-thalamo-cortical pathway (Fig. 1a) [18]. A separate population of inhibitory neurons in the cerebellar nuclei project to the inferior olive, completing a closed-loop circuit of the cerebellar cortex, cerebellar nuclei, and inferior olive [19–21]. Using this nucleo-olivary circuit, the cerebellar cortex can modulate climbing fiber input to Purkinje cells [22–25].

## Event Detection Hypothesis

The low firing frequency of climbing fibers and associated CSs evoked in Purkinje cells prompted the early suggestion that the olivocerebellar system is not capable of encoding information using a conventional rate code. Combining the “phasic” nature of the CS discharge with observations that climbing fibers are highly responsive to small perturbations led to the “event detector” hypothesis [26, 27]. Most of these early experiments were performed in anesthetized or decerebrate preparations. However, during voluntary movements, CS responses to somatosensory stimuli are greatly diminished and instead are evoked when a stimulus is not anticipated, leading to the “unexpected event” hypothesis (for reviews, see [11, 28]). Subsequent work in a variety of preparations and behaviors demonstrated that CSs provide considerable information about reflex and voluntary behaviors and do not just signal events (see Beyond Error Signaling: Parametric and Predictive Encoding). Therefore, the event detector hypothesis failed to capture the complete properties of climbing fibers and their action on Purkinje cells.

## Error Hypothesis

One of the most accepted hypotheses is that CSs signal errors. Initially proposed in the framework of a comparator, Oscarsson postulated that the inferior olive compares command signals from higher centers with feedback from the spinal cord, thereby generating a type of error signal [29]. In support of the comparator hypothesis, the inferior olive integrates both feedforward and feedback information as it receives a variety of excitatory and inhibitory inputs from the spinal cord, nuclei at the mesodiencephalic junction, cerebellar nuclei, and cerebral cortex (for reviews, see [29–31]). However, individual inferior olive neurons generally do not receive both descending and ascending inputs, suggesting that these neurons do not perform the comparison necessary to generate an error signal [30].

The comparator hypothesis quickly evolved into the error hypothesis and was coupled to synaptic plasticity and motor learning (for reviews [32–34]). In the Marr-Albus-Ito hypothesis, motor learning is mediated by long-term depression (LTD) of parallel fiber–Purkinje cell synapses resulting from co-activation of parallel fiber and climbing fiber inputs [35–37]. In this view, CSs are evoked by errors and CSs provide a teaching signal that modifies subsequent SS activity to correct the behavior [37–41]. Although controversial (for reviews, see [34, 42, 43]), for nearly a half century, the error signaling/motor learning hypothesis has dominated the field’s view of climbing fiber function.

**Fig. 2:**
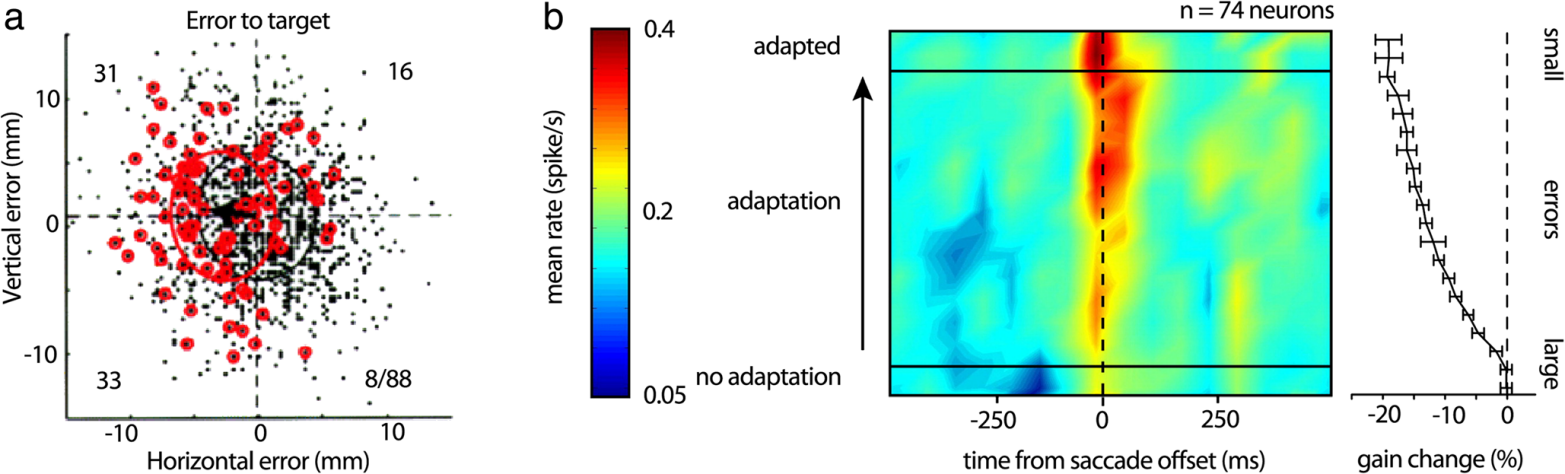
Complex spike firing in relation to errors. **a** Scatterplot of end point position relative to target center for all trials (black dots), CS occurrence trials marked by small red circles, during a reaching task to a target presented on a screen. Total of 88 CS occurred out of 1381 reaches, with the numbers of CSs in each quadrant as indicated. The large ellipses denote the equidistance points (Mahalanobis distance = 1) for each population (red–CS occurring trials, black–all trials). Black arrow illustrates the shift between the centers of the red and black ellipses that quantifies the CSs preferred error direction and defines the CSs error modulation (modified with permission from [41]). **b** Heat map of CS population response from 74 Purkinje cells during an inward saccade adaptation task. Right panel depicts the degree of saccade adaptation, which is inversely related to error magnitude. CS modulation is minimal at the start of the adaptation when errors are maximal and increases as adaptation develops and error magnitude decreases. Maximum CS firing occurs when errors are small (modified with permission from [54])

Many studies observed CS firing in relation to motor errors. In the floccular complex, CSs are driven by retinal-slip during smooth pursuit and VOR adaptation [44–46]. In the ventral paraflocculus, CSs modulate with the retinal slip during the ocular following response [47], and in the oculomotor vermis, CSs modulate with induced saccade errors [48]. The error hypothesis received further support with the observation of CS modulation in relation to reach end point errors in the monkey (Fig. 2a) [41]. In agreement, several arm movement studies documented that CSs modulate with unexpected loads [38], redirection of a reach [49], and during adaptation to visuomotor transformations [50]. In addition, CS discharge increases with perturbations applied during locomotion [51–53].

However, other studies found limited support for the classical error encoding view. As described above, CS discharge is associated with end point errors during saccades. One method to induce saccade end point errors is by changing the instructed target location while the eyes are in flight (Fig. 2b) [54]. Over time, the subject learns to predict the change in target location and alters the motor command so that the eyes successfully reach the desired end point. During this type of saccadic adaptation, the error encoding hypothesis predicts that CSs would be highly modulated early in adaptation, when errors are maximal. As the animal learns to predict the change in target position and errors are reduced, the CS modulation should also decrease. In the oculomotor vermis, however, the opposite relationship is observed, with CS discharge increasing late in adaptation when errors have decreased greatly (Fig. 2b) [54]. A similar build-up of CS discharge occurs as performance errors decrease during smooth pursuit adaptation [55, 56]. These observations demonstrate that CS error signals are conditional upon the behavioral and experimental context and challenge the assumption that climbing fibers predominately respond to motor errors.

Similarly, perturbations and performance errors during reaching in cats do not evoke responses in inferior olive neurons [57]. During reaching movements in the monkey, CS modulation could not be related to direction or speed errors [58, 59] nor were CSs associated with learning a mechanical perturbation [60]. During pseudo-random tracking, CSs are not associated with error events [61]. Smooth pursuit learning does not drive CS modulation in the oculomotor vermis [62]. Also, in the oculomotor vermis, CS error modulation with saccades may be limited to direction errors, and whether they encode error magnitude is unclear [63, 64]. Even when associated with errors, CSs occur with low probability [41, 50, 61]. Therefore, the precision, specificity, and extent to which CSs encode error information remain unknown.

Motor learning does not depend exclusively on climbing fiber input [60, 65–67] nor does it depend solely on LTD at the parallel fiber–Purkinje cell synapse [34]. Also, SS discharge carries robust error signals, both for eye and arm movements [68–72]. Therefore, error processing in the cerebellum is more multi-faceted than originally proposed, and to paraphrase a wise old man from in galaxy far away, “these are not the error signals you are looking for.”

## Rhythmicity and Timing Hypothesis

One of the intriguing properties of the inferior olive neurons is the presence of gap junctions [73, 74]. When combined with a set of voltage-gated calcium and potassium conductances, the electronic coupling supports the synchronous [75–78] and rhythmic activity of most inferior olive neurons (with the exception of neurons in the dorsal cap of Kooy). The 1–10-Hz oscillations occur in both the subthreshold membrane potential [79, 80] and discharge of olivary neurons [81–84]. The timing of the inferior olive neuronal firing is tightly linked to membrane potential phase [85] and the firing waveform reflects the amplitude [86] and synchrony of the subthreshold oscillations [87]. Disruption of the electronic coupling in the inferior olive decreases the coherence of muscle activation [88].

The rhythmicity and synchronicity of climbing fibers suggest a role in movement timing independent of their action on SS firing [12, 84, 89]. Rhythmic CS discharge has been argued to underlie physiological tremor [90]. Elegant studies using simultaneous recordings from multiple Purkinje cells documented these oscillatory and coupled discharge properties of CSs in the intact animal [77, 83, 84, 91], including during rhythmic tongue movements [89]. However, the behavioral studies did not disambiguate the source of the rhythmicity, whether driven by the movements or by the inferior olive.

In the awake, behaving animal, evidence for strong CS rhythmicity remains controversial [11, 92, 93]. The lack of rhythmic CS firing has been particularly striking in the non-human primate [60, 69, 94, 95]. However, CS synchronicity is likely an important element of climbing fiber action in the cerebellar cortex and behavior. For example, the synchronous activation of CF microzones could reliably encode sensory inputs [96] or impose encoding changes on a functionally related group of Purkinje cells [69].

## Control of Purkinje Cell Excitability, Gain Change, and Bistability Hypotheses

The error and motor learning hypotheses do not readily account for spontaneous CS firing and the observation that removal or stimulation of climbing fiber input results in a dramatic change in the SS firing pattern [97–100]. Lesions or blockade of the inferior olive produces large increases in the SS firing rate and stimulation largely decreases. Climbing fiber stimulation suppresses conditioned eye blink responses, suggesting that on-going CSs directly influence motor behavior [100]. Furthermore, lesions of the inferior olive result in the rapid emergence of a striking cerebellar-like motor disorder [101, 102]. Therefore, climbing fiber input must play a role in on-line cerebellar function and motor control.

Several earlier hypotheses on the CS contribution to real-time motor control emphasize short-term changes in Purkinje cell excitability. The “gain change” hypothesis postulated climbing fiber input controls the responses of Purkinje cell inputs, either increasing or decreasing the responsiveness [103, 104]. In the decerebrate preparation, the magnitude of the SS responses to peripheral inputs depends on the timing relative to CS occurrence. A similar gain was observed in the decerebrate ferret during locomotion and led to the “dynamic selection hypothesis” proposing that climbing fibers act to spatially focus a set of Purkinje cell by emphasizing their responsiveness to parallel fiber input [53, 105]. However, in the awake rabbit, SS responses to vestibular and optokinetic stimulation do not appear to be controlled by climbing fiber input [11]. Therefore, while the gain change hypothesis remains interesting, the concept lacks adequate support in intact, behaving animals.

Subsequently, the “bistability” hypothesis stated that CSs control the responses of a Purkinje cell to parallel fiber inputs by switching between “up” and “down” SS firing states [106–108]. In the context of error signaling, the bistability allows for toggling a Purkinje cell between states for instantaneous correction following an error [106]. Other possible functions include providing a short-term memory capabilities or generation of temporal pattern (for review, see [92]). However, CS-coupled changes in SS firing rates are only prominent in reduced or anesthetized preparations. In the awake mouse, togging between high and low SS firing was not observed during optokinetic and vestibular reflexes [92, 93]. In the monkey, short-term changes in SS firing following a CS are limited, with the exception of a brief inactivation period [50, 69, 109]. Several explanations have been offered to account for discrepancies across studies, including Purkinje cell heterogeneity, neuromodulators, and level of network activity [92]. However, in the awake animal, climbing fiber input appears to have a very restricted effect on SS firing rate.

## Complex Spike-Simple Spike Discharge Reciprocity

The discharge of CSs and SSs is often modulated reciprocally in which an increase in CS firing is accompanied by a decrease in SS firing and vice versa. Reciprocal CS–SS modulation is thought to be a fundamental feature of Purkinje cell physiology and its role in controlling movements [98, 110–112]. This reciprocity has been described in several species, stimulus paradigms, and cerebellar cortical regions. Examples include during optokinetic stimulation in the flocculus and paraflocculus of the rabbit [44, 11] and mouse [112, 113] and during smooth pursuit in the monkey [114]. In the cat, CS–SS modulation reciprocity occurs in the vermis during background firing [110, 115], in lobules V and VI following electrical stimulation of the radial and vagus nerve [116], and during passive wrist movements [117]. However, it should be stressed that many of the above reports of CS–SS reciprocity were in reduced or anesthetized preparations.

Whether the reciprocity is due to climbing fiber input was difficult to test due to the tonic action of CSs on SS firing reviewed above [97–100]. A recent study provided clarification using *Ptf1a::cre;Robo3*^*lox/lox*^ mice in which the climbing fibers are selectively re-routed from a contralateral to an ipsilateral projection [112]. Surprisingly, the CS modulation during optokinetic stimulation was reversed as was the SS firing. In addition, climbing fibers acting on inhibitory interneurons likely contribute to the antiphase CS–SS firing pattern, as the interneuron modulation was also inverted.

Reciprocal CS–SS modulation may be less prominent than previously thought. Reciprocity is not present in the dark or during three-dimensional vestibular stimulation [118, 119]. In the oculomotor vermis, reciprocity was not observed for either saccades or smooth pursuit [62]. During pseudorandom tracking, the differences in CS and SS directional tuning are distributed uniformly [61]. A similar diversity in CS and SS tuning occurs in reaching tasks [41, 50, 59]. Therefore, CS and SS discharge reciprocity appears to be conditional rather than deterministic.

## Beyond Classical Concepts: Parametric and Predictive Encoding

**Fig. 3:**
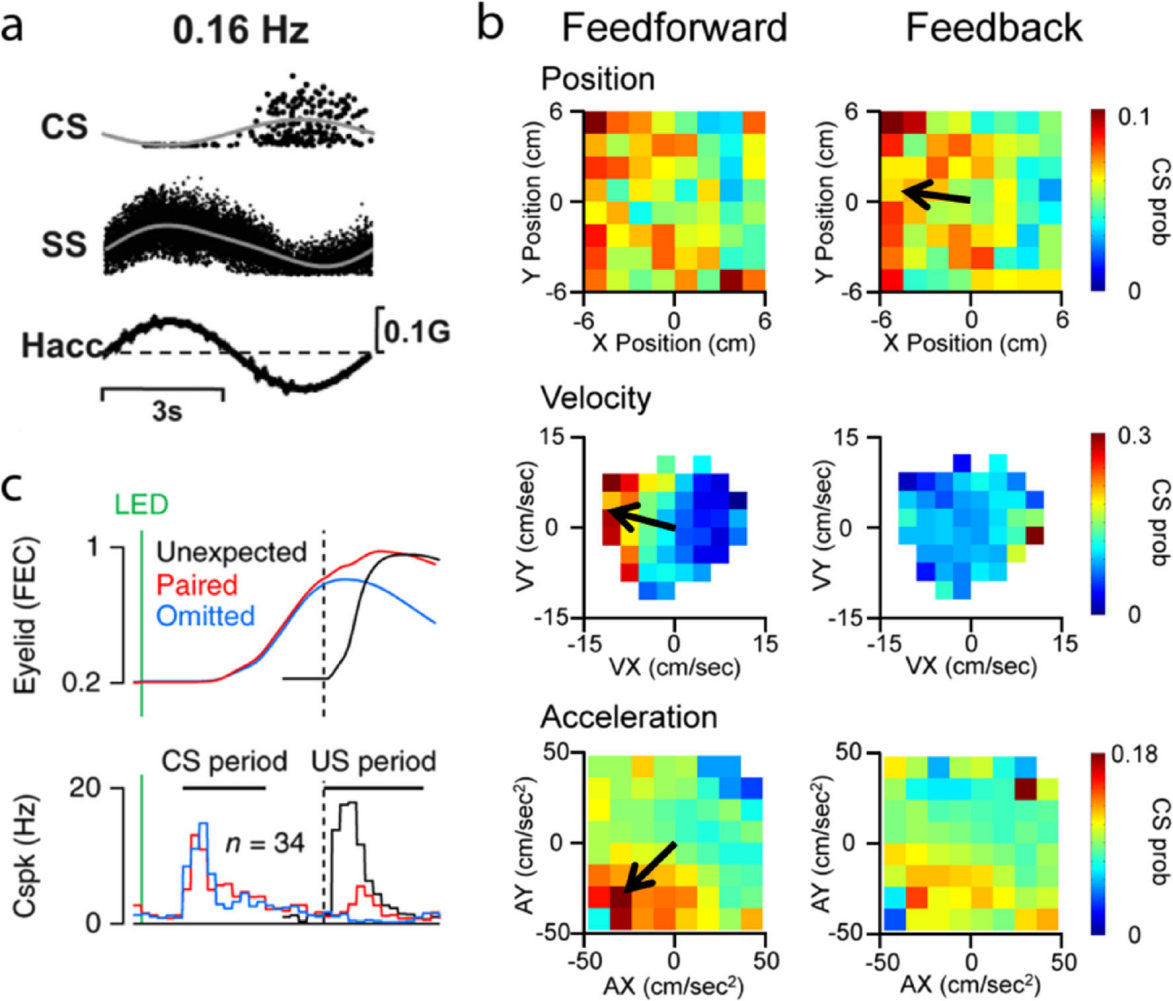
Complex spike modulation with movement parameters. **a** Example of CS and SS firing relative to head acceleration (Hacc) during lateral translation at 0.16 Hz (modified with permission from [118]). **b** Feedforward and feedback CS probability maps (CS prob) with hand position (X, Y), velocity (VX, VY), and acceleration (AX, AY) during pseudo-random tracking, each map obtained from a different cell (modified with permission from [61]). The modulation significance of each firing plot is indicated by the directional tuning vectors (black arrows). **c** Top panel shows average eyelid responses to periocular airpuffs (unexpected–black, paired with conditioning LED cue–red, conditioning LED cue only–blue, green vertical bar indicates conditioning LED cue). Bottom panel demonstrates CS modulation preceding the airpuff in the conditioning stimulus period (modified with permission from [123])

During various vestibular and oculomotor behaviors, CSs carry parametric information about movements. For example, CS modulation occurs in the flocculus during VOR rotation in the dark when retinal slip is absent [119], carries kinematic information, in addition to retinal slip signals, in the ventral paraflocculus during ocular pursuit [47], and is directionally tuned in the nodulus during three-dimensional vestibular stimulation (Fig. 3a) [118]. Also, CS firing modulates with both reach direction and amplitude [58]. A relationship to motor commands has been suggested based on the increased CS discharge around limb movement onset [58, 109] and climbing fiber modulation with vibrissae and tongue movements [58, 109].

Previous studies typically examined CS modulation during low dimensional, stereotypic movements, including saccades, VOR, and reaching. Therefore, we evaluated CS firing during a pseudo-random, manual tracking task in the monkey [61]. Pseudo-random tracking allows for an examination of the interactions among CS discharge and behavior in which the correlations between parameters are reduced and provides extensive coverage of the work space [68, 120]. The result is a robust data set to assess the behavioral parameters that modulate climbing fiber input. Using reverse correlation, we constructed both feedforward and feedback two-dimensional probability maps of CS firing with kinematics (hand position, velocity, and acceleration) and with position error, a measure of tracking performance (Fig. 3b).

For Purkinje cells in lobules V and VI, CSs significantly encode all three kinematic parameters and position error in this task (Fig. 3b). The CSs are spatially tuned and provide a linear representation of each parameter. Modulation with acceleration is particularly common. The paradigm also provides a definition of salient events, for example an error event defined as crossing out of the target, as this triggers the need for a timely corrective action. During pseudo-random tracking, climbing fiber modulation was not related to “events”, either for position error or kinematics. Therefore, CSs carry an array of continuous parametric motor signals and support the hypothesis that climbing fiber input has a prominent role in online motor control.

In addition, increasing evidence challenges the concept that climbing fiber input is solely feedback driven. For example, CSs modulate in response to inferred errors related to eye movements [119, 121, 122]. During pseudo-random tracking, feedforward CS modulation is almost three times more common than feedback modulation (Fig. 3b) [61]. Highlighting the predictive nature of climbing fiber input, CSs precede but rarely respond to errors. Feedforward CS responses occur during eye blink conditioning, with CS increases prior to and predicting the conditioned response (Fig. 3c) [123, 124].

The mechanism underlying predictive CS encoding remains to be determined. As noted above, the inferior olive receives a variety of excitatory and inhibitory inputs from multiple structures that provide both feedforward and feedback information. For the development of predictive CS signals during classical conditioning, recurrent activity within the olivocerebellar network was postulated to play a major role [124]. Other potential sources of feedforward signals are the premotor and motor cortices. Intriguingly, the responses of inferior olivary neurons to glutamatergic inputs from the motor cortex are bi-phasic in a manner that appears to penalize late inputs [125]. This pattern could create a bias toward feedforward motor signals and a mechanism for predictive CS modulation. Taken together, these observations demonstrate that CS discharge contains predictive motor signals about multiple aspects of the upcoming behavior, instead of only reporting errors or providing only sensory feedback.

## Dynamic Encoding Hypothesis

Error and motor learning roles for climbing fiber action in the cerebellum have dominated the literature since their introduction. This review has highlighted several problems with these views. Clearly, CSs do not simply signal errors, as they fail to respond with errors in many behaviors and instead modulate predictively with multiple parameters of movement. Movements with higher dimensionality reveal the predictive nature of CS discharge. Therefore, CS modulation with behavior is highly dependent on the experimental paradigm, further raising the need to consider other functions for climbing fiber input.

Two additional features of climbing fiber input and CS discharge lead us to consider other possible functions. First, CSs fire spontaneously and the importance of that background firing to SS firing and cerebellar function implies an ongoing role in cerebellar computations, irrespective of errors or learning. Second, the massive depolarization of the Purkinje cell due to climbing fiber input likely resets the residual effects of prior inputs as well as change how subsequent inputs act on the Purkinje cell (Fig. 1c). Therefore, we hypothesized that climbing fibers change the encoding of the information in the SS firing.

**Fig. 4:**
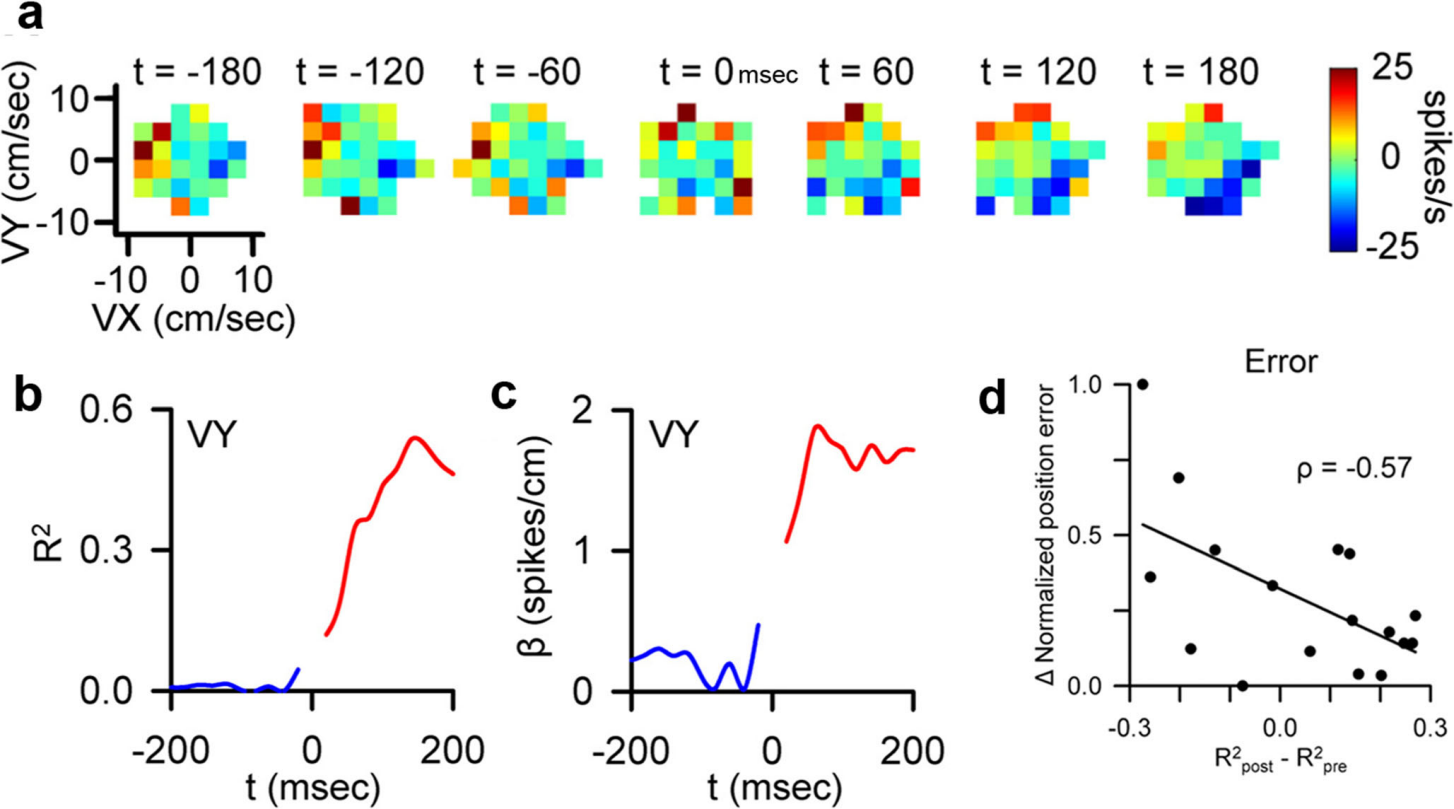
Complex spike-coupled changes in SS encoding. **a** Purkinje cell SS firing maps (mean-subtracted) with hand velocity (VX and VY) reveal an increased modulation with VY after CS occurrence (*t* = 0 msec). The SS modulation with each parameter before and after the CS discharge was quantified with a regression analysis in which the coefficient of determination, *R*^2^, provides a measure of SS encoding strength and the regression coefficient, β, a measure of SS sensitivity (see Methods in [69]). **b** Plotted for this example Purkinje neuron is the pre- (blue trace) and post-CS (red trace) *R*^2^ plot that highlights the increase in SS modulation with VY following CS occurrence. Note the step changes between blue and red traces. **c** Plot of pre- (blue trace) and post-CS (red trace) SS sensitivity relative to VY demonstrates a corresponding increase after CS occurrence. Plots in **b** and **c** from the same cell shown in **a**. **d** Plot of change in position error relative to CS occurrence (post-pre) with the strength (defined by *R*^2^) of CS-coupled changes in SS encoding for the population of Purkinje cells. Magnitude of position error decreases as the SS encoding of error increases (modified with permission from [69])

We evaluated this hypothesis during pseudo-random tracking, revealing that climbing fiber discharge dynamically controls the information present in the SS firing, triggering robust and rapid changes in SS encoding of motor signals in Purkinje cells [69]. The changes in encoding consist of increases or decreases in the SS sensitivity to kinematics or position errors. An example of the change in SS encoding is shown in Fig. 4 for a Purkinje cell with SS modulation with hand velocity. Prior to CS occurrence, the encoding of velocity in the SS discharge is weak and markedly increases following CS occurrence (Fig. 4a–c). Furthermore, the changes in encoding are tightly coupled to CS occurrence. In the light of the gain change, bistability, and rhythmicity hypotheses, we investigated and showed that encoding changes are not due to differences in SS firing rates or variability. Nor are the changes in sensitivity due to CS rhythmicity, as there was no evidence for rhythmicity of CS firing during pseudorandom tracking.

In addition, the CS-coupled changes in encoding are not evoked by changes in kinematics or position errors. Instead, CS discharge most often leads alterations in behavior, consistent with our recent report [61]. Nearly all of the CS-coupled changes in SS encoding changes are associated with predictive CS modulation with behavior. Furthermore, at the population level, the changes in sensitivity are consistent with optimizing behavior. Increases in SS encoding of position error are followed by and scale with decreases in error (Fig. 4d). Also, increases in SS encoding of a kinematic parameter are associated with larger changes in that parameter than are decreases in SS encoding. Intriguingly, the CS-coupled changes in encoding for a given Purkinje cell tend to oppose the encoding drift occurring independent of CS firing. For example, a Purkinje cell with a CS-coupled increase in SS encoding of velocity will tend to show a decrease in velocity encoding in the absence of CSs.

An outstanding question is the potential mechanism(s) by which these alterations in SS encoding occur. A number of candidates could explain the changes. First, the number of spikes in a given climbing fiber discharge affects the CS burst pattern, dendritic Ca^2+^ spikes, and plasticity [86, 126]. Just as the degree of parallel fiber–Purkinje cell synaptic plasticity varies with the duration of the CS discharge [39, 127], short-term changes in firing have been observed. In anesthetized rats, increased SS firing preceding climbing fiber discharge is followed by a higher number of CS spikelets that in turn associated with a subsequent reduction in SS activity [128]. An additional mechanism for CS-coupled changes in SS encoding is via local inhibition by GABAergic interneurons that modifies the conductance changes and Ca^2+^ fluxes evoked by climbing fiber input [129, 130]. Low amplitude, extended Ca^2+^ responses inhibit the parallel fiber–Purkinje cell synapse, while high amplitude Ca^2+^ fluxes could increase the gain, potentially facilitating bidirectional changes in SS encoding [131, 132]. Also, the timing of climbing fiber discharge may differentially modulate parallel fiber input and contribute to the direction of synaptic potentiation [133, 134].

**Fig. 5:**
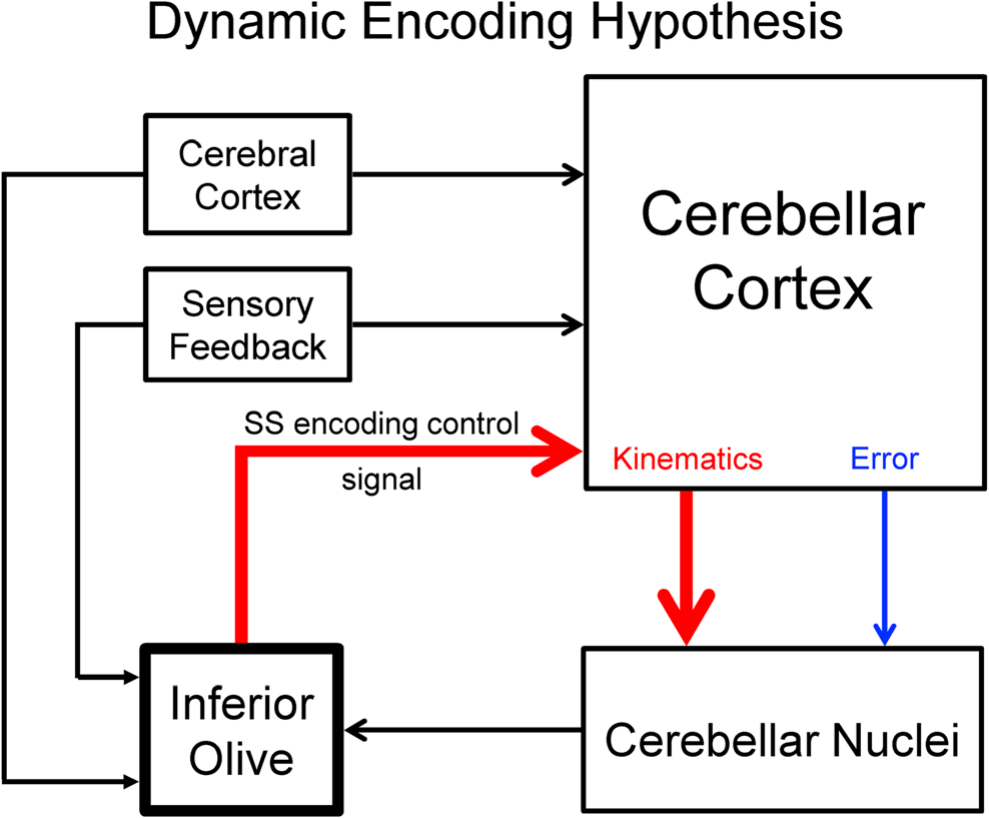
Illustration of the dynamic encoding hypothesis. Inferior olive integrates behavioral information from the cerebral cortex, sensory system, and computational state of the cerebellar cortex from the cerebellar nuclei. Cerebellar cortex also receives cortical, brainstem (not shown), and sensory information and transforms these inputs into representations of movement kinematics (red arrow) and motor errors (blue arrow) in the simple spike firing of Purkinje cells. The dynamic encoding hypothesis states that climbing fibers, the output of the inferior olive, provide an encoding control signal (red arrow) that resets the sensitivity of the Purkinje cell simple spike firing. Cerebellar cortex output is integrated at level of the cerebellar nuclei. In this example, inferior olive activity drives an increase in simple spike encoding of kinematics as indicated by increased thickness of red lines (similar to Fig. 4 example). Climbing fiber can also drive decreases in kinematic encoding, as well as bidirectional changes in error encoding

Integrating these new observations with previous results argues for a new hypothesis in which climbing fiber discharge dynamically controls the information present in the SS discharge (Fig. 5). Purkinje cells have the capacity for a large theoretical bandwidth [135], and an array of movement signals is encoded in the SS firing (for review, see [136]). Both climbing fiber input and Purkinje cell output are crucial for continuous, online control of movement as well as motor learning and adaptation. The dynamic encoding hypothesis provides a framework for both spontaneous and evoked climbing fiber discharge. In this view, spontaneous climbing fiber input resets the encoding state of the Purkinje cell to maintain an optimal computational state of the SS firing. In other words, the CS corrects for continuous “drifts” in SS encoding, stabilizing the information conveyed by the Purkinje cell output (Fig. 5). Conversely, behaviorally evoked climbing fiber discharge, either predictive or feedback driven, reallocates the bandwidth of the Purkinje cell to optimize the SS information to the most salient aspects of the task to control behavior (Fig. 5).

A major challenge in determining the function of climbing fiber discharge is interpreting the spectrum of experimental observations regarding spontaneous and evoked CS firing. Importantly, the dynamic encoding hypothesis is compatible with most previous results. The same behavioral parameters linearly modulate both CSs and SSs in the same reference frames [61, 68, 70, 137]. This suggests that these two discharge modes of Purkinje cells function in concert during movements, as opposed to acting independently. In agreement, Purkinje cells are organized according to CS directional tuning such that the SS population response, when based on climbing fiber directional responsiveness, improves the decoding the speed and direction of saccades [138]. Also, the parasagittal organization of the olivocerebellar projection and tendency for synchronous climbing fiber activation will work to reset the SS representations within a microzone and effect a coordinated change of the information to downstream targets.

Recent studies emphasize that the heterogeneity in the cerebellar circuity is associated with differential Purkinje cell excitability and firing statistics. For example, the spontaneous SS and CS firing rates are higher in zebrin II − zones than in zebrin II + zones [139, 139–141]. Zebrin II + zones also have higher SS variability and longer duration SS suppression following a CS [139]. In addition, Purkinje cells in zebrin II ± bands respond differently to PF input [142, 143] and show different SS modulation patterns preceding CS occurrence [144]. In our study, the CS-coupled encoding changes occur in the vast majority of Purkinje cells recorded, arguing that the encoding is not necessarily restricted to specific subpopulations defined by the local heterogeneity of the cerebellar cortex. However, it remains to be determined to what degree local differences in the circuitry modulate the dynamic encoding processes.

For CS signaling events or errors, the evoked climbing fiber discharge reflects the need to change the encoding state of Purkinje cells due to errors. The changes in SS sensitivity evoked by CS are arguably a form of bistability. Given that bistability occurs primarily in reduced or anesthetized preparations in which the physiology of both Purkinje cells and the cerebellar cortical circuitry is altered (for review, see [92]), the information present in SS firing is limited. Therefore, the effect of a CS manifests as an overall change in firing rate as opposed to a change in encoding. Finally, the dynamic encoding hypothesis accounts for the suppressive action of climbing fibers on SS firing. As uncorrected drifts in SS encoding accumulate and firing rates increase, cerebellar representations of motor behavior are corrupted and ataxia develops. Thus, this novel framework provides a unifying view of climbing fiber function, capable of incorporating previous experimental observations.

Importantly, the dynamic encoding concept provides a unifying framework for both spontaneous and evoked CSs. The hypothesis is also compatible with climbing fiber involvement in learning and adaptation [32–34]. For example, spontaneous climbing fiber input has been proposed to perturb movements as a probe for initiating plasticity [145] and CS-evoked changes in SS encoding are consistent with this view. Behaviorally evoked CSs are likely to engage plasticity mechanisms at the parallel fiber–Purkinje cell synapses within a microzone. Together with previous descriptions of bi-directional plasticity mechanisms [133, 134, 146, 147], SS resetting may contribute to the rules governing the direction of plasticity.

## Conclusions

This review examined the various hypotheses on the function of the climbing fiber–Purkinje cell synapse. Each of the traditional hypotheses has merit and has problems. Newer results show that the field needs to move beyond CSs serving as an error feedback signal. Climbing fiber input has spatially rich information about kinematics and performance errors present. We postulate that climbing fiber discharge controls the signals encoded in the SS firing. This dynamic encoding hypothesis is consistent with many of the observations in the literature.
